# 4-Chloro-*N*′-[(*Z*)-4-(dimethyl­amino)benzyl­idene]benzohydrazide mono­hydrate

**DOI:** 10.1107/S1600536808022861

**Published:** 2008-07-26

**Authors:** Hoong-Kun Fun, P. S. Patil, Samuel Robinson Jebas, K. V. Sujith, B. Kalluraya

**Affiliations:** a X-ray Crystallography Unit, School of Physics, Universiti Sains Malaysia, 11800 USM, Penang, Malaysia; b Department of Studies in Physics, Mangalore University, Mangalagangotri, Mangalore 574 199, India; c Department of Studies in Chemistry, Mangalore University, Mangalagangotri, Mangalore 574 199, India

## Abstract

In the title compound, C_16_H_16_ClN_3_O·H_2_O, the dihedral angle between the two aromatic rings is 44.58 (11)°. The N atom of the dimethyl­amino group adopts a pyramidal configuration. In the crystal structure, mol­ecules are linked into a two-dimensional network parallel to the (001) plane by inter­molecular N—H⋯O, O—H⋯N and O—H⋯O hydrogen bonds involving the water mol­ecule and C—H⋯Cl hydrogen bonds. In addition, C—H⋯π inter­actions are observed.

## Related literature

For the biological activities of hydrazones, see: Bedia *et al.* (2006 ▶); Rollas *et al.* (2002 ▶); Terzioglu & Gürsoy (2003 ▶); Duraisamy *et al.* (2008 ▶); Singh *et al.* (1992 ▶); Ergenç & Günay, (1998 ▶); Durgun *et al.* (1993 ▶). For bond-length data, see: Allen *et al.* (1987 ▶).
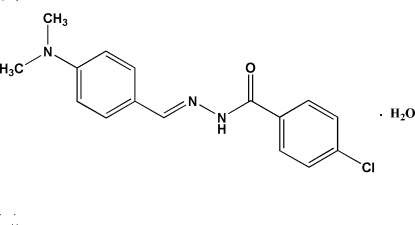


## Experimental

### 

#### Crystal data

C_16_H_16_ClN_3_O·H_2_O
*M* *_r_* = 319.78
Orthorhombic, 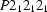

*a* = 6.4418 (1) Å
*b* = 6.9344 (1) Å
*c* = 33.8083 (7) Å
*V* = 1510.22 (4) Å^3^
*Z* = 4
Mo *K*α radiation
μ = 0.26 mm^−1^
*T* = 100.0 (1) K
0.32 × 0.16 × 0.07 mm

#### Data collection

Bruker SMART APEXII CCD area-detector diffractometer
Absorption correction: multi-scan (*SADABS*; Bruker, 2005 ▶) *T*_min_ = 0.921, *T*_max_ = 0.981
12336 measured reflections
4311 independent reflections
3301 reflections with *I* > 2σ(*I*)
*R*_int_ = 0.056

#### Refinement

*R*[*F* ^2^ > 2σ(*F* ^2^)] = 0.050
*wR*(*F* ^2^) = 0.114
*S* = 1.01
4311 reflections
205 parameters
2 restraints
H atoms treated by a mixture of independent and constrained refinement
Δρ_max_ = 0.34 e Å^−3^
Δρ_min_ = −0.34 e Å^−3^
Absolute structure: Flack (1983 ▶), 1724 Friedel pairs
Flack parameter: −0.14 (7)

### 

Data collection: *APEX2* (Bruker, 2005 ▶); cell refinement: *APEX2*; data reduction: *SAINT* (Bruker, 2005 ▶); program(s) used to solve structure: *SHELXTL* (Sheldrick, 2008 ▶); program(s) used to refine structure: *SHELXTL*; molecular graphics: *SHELXTL*; software used to prepare material for publication: *SHELXTL* and *PLATON* (Spek, 2003 ▶).

## Supplementary Material

Crystal structure: contains datablocks global, I. DOI: 10.1107/S1600536808022861/ci2637sup1.cif

Structure factors: contains datablocks I. DOI: 10.1107/S1600536808022861/ci2637Isup2.hkl

Additional supplementary materials:  crystallographic information; 3D view; checkCIF report

## Figures and Tables

**Table 1:** Hydrogen-bond geometry (Å, °) *Cg*1 and *Cg*2 are the centroids of the C1–C6 and C9–C14 rings, respectively.

*D*—H⋯*A*	*D*—H	H⋯*A*	*D*⋯*A*	*D*—H⋯*A*
O1*W*—H1*W*1⋯N3^i^	0.84	2.28	3.045 (2)	152
N1—H1*N*1⋯O1*W*	0.86 (1)	2.00 (1)	2.843 (3)	169 (2)
O1*W*—H2*W*1⋯O1^ii^	0.84	2.02	2.790 (2)	152
O1*W*—H2*W*1⋯N2^ii^	0.84	2.59	3.240 (3)	135
C15—H15*C*⋯Cl1^iii^	0.96	2.78	3.704 (2)	163
C1—H1⋯*Cg*1^iv^	0.93	2.97	3.621 (2)	128
C4—H4⋯*Cg*1^v^	0.93	2.89	3.565 (2)	130
C10—H10⋯*Cg*2^vi^	0.93	2.87	3.589 (2)	135
C13—H13⋯*Cg*2^vii^	0.93	2.81	3.497 (3)	131

Symmetry codes: (i) 
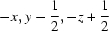
; (ii) 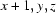; (iii) 
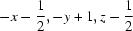
; (iv) 
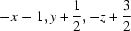
; (v) 
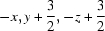
; (vi) 
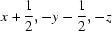
; (vii) 
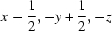
.

**Fig. 1.**
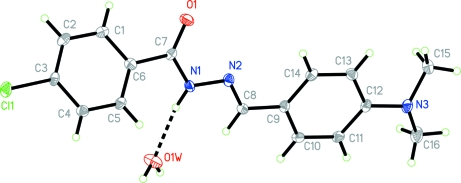
The molecular structure of the title compound, showing 50% probability displacement ellipsoids and the atom-numbering scheme.

**Fig. 2.**
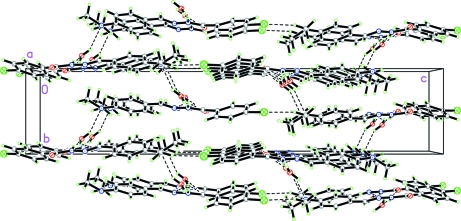
The crystal packing of the title compound, viewed along the a axis.

## supplementary crystallographic information

### Comment

Hydrazone compounds have been demonstrated to possess antimicrobial, anticonvulsant, analgesic, antiinflammatory, antiplatelet, antitubercular, anticancer and antitumoral activities (Bedia *et al.*, 2006; Rollas *et al.*, 2002; Terzioglu & Gürsoy, 2003). They are used as chromogenic receptors to show colorimetric responses and UV-Vis spectral changes in the presence of fluoride ions in organic solvents (Duraisamy *et al.*, 2008). Hydrazide-hydrazone compounds are not only intermediates but they are also very effective organic compounds in their own right. When they are used as intermediates, coupling products can be synthesized by using the active hydrogen component of CONHNCH azometine group (Singh *et al.*, 1992). *N*-Alkyl hydrazides can be synthesized by reduction of hydrazones with NaBH_4_ (Ergenç & Günay, 1998) and substituted 1,3,4-oxadiazolines can be synthesized when hydrazones are heated in the presence of acetic anhydride (Durgun *et al.*, 1993). Prompted by these reviews, the title compound was synthesized and its crystal structure reported.

The bond lengths and angles in the title molecule (Fig. 1) are found to have normal values (Allen *et al.*, 1987). The dihedral angle between the two benzene rings (C1–C6 and C9–C14) is 44.58 (11)° indicating that the molecule is non-planar. Atom N3 adopts a pyramidal configuration.

The crystal packing (Fig. 2) shows that the molecules are linked into two-dimensional networks parallel to the (001) plane by intermolecular O—H···N, O—H···O and C—H···Cl hydrogen bonds. In addition, the packing is stabilized by C—H···π interactions.

### Experimental

The title compound was prepared by Schiff base condensation of 4-chlorophenyl hydrazide (0.01 mol) and 4-(dimethylamino)benzaldehyde (0.01 mol) in ethanol (30 ml) with 3 drops of concentrated H_2_SO_4_. Excess ethanol was removed from the reaction mixture under reduced pressure. The solid product obtained was filtered, washed with water and dried. Single crystals suitable for X-ray analysis were obtained from an ethanol solution by slow evaporation (yield 64%). Analysis % for C_16_H_16_N_3_OCl found (calculated): C 63.62 (63.68), H 5.37 (5.3), N 13.88 (13.93).

### Refinement

The imino H atom was located in a difference map and refined with a N-H distance restraint of 0.85 (1) Å. The water H atoms were also located in a difference map and allowed to ride on the O atom, with U_iso_ = 1.5U_eq_(O). The remaining H atoms were positioned geometrically [C-H = 0.93-0.96 %A] and refined using a riding model, with *U*_iso_(H) = 1.2*U*_eq_(C) and 1.5U_eq_(C_methyl_). A rotating group model was used for the methyl groups.

### Crystal data

**Table:**

C_16_H_16_ClN_3_O·H_2_O	*F*_000_ = 672
*M**_r_* = 319.78	*D*_x_ = 1.406 Mg m^−^^3^
Orthorhombic, *P*2_1_2_1_2_1_	Mo *K*α radiation λ = 0.71073 Å
Hall symbol: P 2ac 2ab	Cell parameters from 1906 reflections
*a* = 6.4418 (1) Å	θ = 2.4–23.3º
*b* = 6.9344 (1) Å	µ = 0.26 mm^−^^1^
*c* = 33.8083 (7) Å	*T* = 100.0 (1) K
*V* = 1510.22 (4) Å^3^	Needle, colourless
*Z* = 4	0.32 × 0.16 × 0.07 mm

### Data collection

**Table:**

Bruker SMART APEXII CCD area-detector diffractometer	4311 independent reflections
Radiation source: fine-focus sealed tube	3301 reflections with *I* > 2σ(*I*)
Monochromator: graphite	*R*_int_ = 0.056
*T* = 100.0(1) K	θ_max_ = 30.1º
φ and ω scans	θ_min_ = 2.4º
Absorption correction: multi-scan(SADABS; Bruker, 2005)	*h* = −9→9
*T*_min_ = 0.921, *T*_max_ = 0.981	*k* = −9→6
12336 measured reflections	*l* = −47→37

### Refinement

**Table:**

Refinement on *F*^2^	Hydrogen site location: inferred from neighbouring sites
Least-squares matrix: full	H atoms treated by a mixture of independent and constrained refinement
*R*[*F*^2^ > 2σ(*F*^2^)] = 0.050	*w* = 1/[σ^2^(*F*_o_^2^) + (0.0492*P*)^2^] where *P* = (*F*_o_^2^ + 2*F*_c_^2^)/3
*wR*(*F*^2^) = 0.114	(Δ/σ)_max_ = 0.001
*S* = 1.01	Δρ_max_ = 0.34 e Å^−^^3^
4311 reflections	Δρ_min_ = −0.34 e Å^−^^3^
205 parameters	Extinction correction: none
2 restraints	Absolute structure: Flack (1983), 1724 Friedel pairs
Primary atom site location: structure-invariant direct methods	Flack parameter: −0.14 (7)
Secondary atom site location: difference Fourier map	

### Special details

**Table:**

Experimental. The data was collected with the Oxford Cyrosystem Cobra low-temperature attachment.
Geometry. All e.s.d.'s (except the e.s.d. in the dihedral angle between two l.s. planes) are estimated using the full covariance matrix. The cell e.s.d.'s are taken into account individually in the estimation of e.s.d.'s in distances, angles and torsion angles; correlations between e.s.d.'s in cell parameters are only used when they are defined by crystal symmetry. An approximate (isotropic) treatment of cell e.s.d.'s is used for estimating e.s.d.'s involving l.s. planes.
Refinement. Refinement of *F*^2^ against ALL reflections. The weighted *R*-factor *wR* and goodness of fit *S* are based on *F*^2^, conventional *R*-factors *R* are based on *F*, with *F* set to zero for negative *F*^2^. The threshold expression of *F*^2^ > σ(*F*^2^) is used only for calculating *R*-factors(gt) *etc*. and is not relevant to the choice of reflections for refinement. *R*-factors based on *F*^2^ are statistically about twice as large as those based on *F*, and *R*- factors based on ALL data will be even larger.

### Fractional atomic coordinates and isotropic or equivalent isotropic displacement parameters (Å^2^)

**Table:**

	*x*	*y*	*z*	*U*_iso_*/*U*_eq_	
Cl1	0.39865 (9)	0.50981 (9)	0.568340 (15)	0.02126 (14)	
O1	−0.3268 (2)	0.4990 (2)	0.42597 (4)	0.0206 (3)	
N1	−0.0370 (3)	0.4579 (3)	0.38853 (5)	0.0146 (4)	
N2	−0.1527 (3)	0.4602 (3)	0.35420 (5)	0.0151 (4)	
N3	−0.4409 (3)	0.4624 (3)	0.17091 (5)	0.0166 (4)	
C1	−0.0827 (4)	0.4376 (3)	0.49549 (6)	0.0154 (5)	
H1	−0.2199	0.3967	0.4971	0.018*	
C2	0.0388 (4)	0.4421 (3)	0.52925 (6)	0.0173 (5)	
H2	−0.0147	0.4008	0.5534	0.021*	
C3	0.2405 (3)	0.5086 (4)	0.52666 (6)	0.0154 (4)	
C4	0.3243 (4)	0.5704 (3)	0.49095 (6)	0.0157 (5)	
H4	0.4595	0.6170	0.4897	0.019*	
C5	0.2028 (3)	0.5613 (3)	0.45730 (7)	0.0153 (5)	
H5	0.2578	0.6005	0.4332	0.018*	
C6	−0.0002 (3)	0.4943 (4)	0.45901 (6)	0.0148 (4)	
C7	−0.1372 (3)	0.4843 (3)	0.42347 (6)	0.0136 (4)	
C8	−0.0579 (4)	0.4106 (3)	0.32251 (6)	0.0147 (5)	
H8	0.0795	0.3699	0.3239	0.018*	
C9	−0.1631 (4)	0.4172 (3)	0.28408 (6)	0.0135 (5)	
C10	−0.0582 (4)	0.3550 (3)	0.25039 (7)	0.0158 (5)	
H10	0.0757	0.3062	0.2527	0.019*	
C11	−0.1513 (4)	0.3651 (3)	0.21331 (6)	0.0156 (5)	
H11	−0.0797	0.3200	0.1913	0.019*	
C12	−0.3506 (4)	0.4420 (3)	0.20852 (6)	0.0152 (5)	
C13	−0.4564 (3)	0.5034 (4)	0.24292 (6)	0.0160 (4)	
H13	−0.5897	0.5538	0.2408	0.019*	
C14	−0.3643 (3)	0.4893 (3)	0.27960 (6)	0.0151 (4)	
H14	−0.4376	0.5286	0.3019	0.018*	
C15	−0.6672 (4)	0.4541 (4)	0.16837 (7)	0.0205 (5)	
H15A	−0.7266	0.5409	0.1874	0.031*	
H15B	−0.7132	0.3251	0.1738	0.031*	
H15C	−0.7105	0.4907	0.1423	0.031*	
C16	−0.3386 (4)	0.3595 (4)	0.13815 (7)	0.0245 (6)	
H16A	−0.1993	0.4065	0.1352	0.037*	
H16B	−0.4144	0.3811	0.1141	0.037*	
H16C	−0.3354	0.2239	0.1438	0.037*	
H1N1	0.0892 (19)	0.419 (3)	0.3882 (7)	0.028 (7)*	
O1W	0.3779 (3)	0.3316 (2)	0.37632 (5)	0.0259 (4)	
H1W1	0.4093	0.2594	0.3573	0.039*	
H2W1	0.4830	0.3923	0.3837	0.039*	

### Atomic displacement parameters (Å^2^)

**Table:**

	*U*^11^	*U*^22^	*U*^33^	*U*^12^	*U*^13^	*U*^23^
Cl1	0.0253 (3)	0.0248 (3)	0.0137 (3)	−0.0021 (3)	−0.0051 (2)	0.0007 (3)
O1	0.0130 (7)	0.0297 (9)	0.0192 (8)	0.0003 (7)	−0.0006 (6)	−0.0030 (8)
N1	0.0122 (9)	0.0197 (11)	0.0120 (9)	0.0011 (8)	−0.0031 (7)	−0.0003 (8)
N2	0.0170 (9)	0.0155 (10)	0.0127 (9)	−0.0013 (7)	−0.0028 (7)	−0.0004 (7)
N3	0.0203 (10)	0.0189 (11)	0.0107 (9)	0.0014 (8)	−0.0012 (7)	−0.0007 (8)
C1	0.0170 (11)	0.0118 (11)	0.0174 (11)	0.0005 (8)	0.0039 (9)	−0.0007 (8)
C2	0.0236 (12)	0.0168 (12)	0.0117 (11)	−0.0016 (9)	0.0041 (9)	−0.0012 (9)
C3	0.0221 (11)	0.0160 (11)	0.0081 (10)	0.0016 (10)	−0.0016 (8)	−0.0010 (10)
C4	0.0149 (11)	0.0166 (12)	0.0155 (12)	−0.0004 (8)	0.0005 (9)	−0.0013 (9)
C5	0.0193 (12)	0.0157 (12)	0.0110 (11)	0.0009 (8)	0.0017 (9)	−0.0003 (9)
C6	0.0151 (10)	0.0148 (11)	0.0144 (10)	0.0012 (9)	−0.0001 (8)	−0.0042 (10)
C7	0.0170 (10)	0.0132 (11)	0.0108 (10)	−0.0012 (9)	0.0007 (8)	0.0003 (9)
C8	0.0124 (10)	0.0162 (11)	0.0155 (12)	−0.0015 (9)	−0.0007 (9)	0.0006 (9)
C9	0.0157 (11)	0.0128 (11)	0.0121 (11)	−0.0013 (8)	−0.0013 (9)	0.0004 (8)
C10	0.0131 (11)	0.0179 (12)	0.0164 (12)	−0.0009 (9)	0.0025 (9)	−0.0008 (9)
C11	0.0166 (11)	0.0191 (12)	0.0111 (11)	−0.0002 (9)	0.0025 (9)	−0.0030 (9)
C12	0.0192 (11)	0.0127 (11)	0.0136 (11)	−0.0016 (9)	−0.0013 (9)	0.0021 (8)
C13	0.0145 (10)	0.0169 (11)	0.0167 (11)	0.0024 (10)	0.0000 (8)	−0.0003 (10)
C14	0.0196 (10)	0.0142 (11)	0.0116 (10)	0.0002 (10)	0.0022 (8)	−0.0025 (9)
C15	0.0207 (12)	0.0222 (14)	0.0186 (12)	0.0021 (10)	−0.0054 (9)	0.0008 (10)
C16	0.0297 (14)	0.0304 (15)	0.0134 (12)	0.0070 (11)	−0.0010 (10)	−0.0015 (10)
O1W	0.0162 (9)	0.0304 (10)	0.0310 (10)	0.0004 (7)	−0.0004 (8)	−0.0137 (8)

### Geometric parameters (Å, °)

**Table:**

Cl1—C3	1.739 (2)	C8—C9	1.466 (3)
O1—C7	1.229 (2)	C8—H8	0.93
N1—C7	1.358 (3)	C9—C10	1.393 (3)
N1—N2	1.379 (2)	C9—C14	1.397 (3)
N1—H1N1	0.857 (10)	C10—C11	1.391 (3)
N2—C8	1.280 (3)	C10—H10	0.93
N3—C12	1.405 (3)	C11—C12	1.400 (3)
N3—C15	1.461 (3)	C11—H11	0.93
N3—C16	1.473 (3)	C12—C13	1.414 (3)
C1—C2	1.384 (3)	C13—C14	1.378 (3)
C1—C6	1.399 (3)	C13—H13	0.93
C1—H1	0.93	C14—H14	0.93
C2—C3	1.382 (3)	C15—H15A	0.96
C2—H2	0.93	C15—H15B	0.96
C3—C4	1.390 (3)	C15—H15C	0.96
C4—C5	1.382 (3)	C16—H16A	0.96
C4—H4	0.93	C16—H16B	0.96
C5—C6	1.389 (3)	C16—H16C	0.96
C5—H5	0.93	O1W—H1W1	0.84
C6—C7	1.493 (3)	O1W—H2W1	0.83
			
C7—N1—N2	118.25 (17)	C10—C9—C14	118.2 (2)
C7—N1—H1N1	120.4 (17)	C10—C9—C8	119.4 (2)
N2—N1—H1N1	120.3 (17)	C14—C9—C8	122.4 (2)
C8—N2—N1	116.33 (19)	C11—C10—C9	120.8 (2)
C12—N3—C15	117.51 (19)	C11—C10—H10	119.6
C12—N3—C16	116.49 (19)	C9—C10—H10	119.6
C15—N3—C16	112.54 (19)	C10—C11—C12	121.3 (2)
C2—C1—C6	120.4 (2)	C10—C11—H11	119.4
C2—C1—H1	119.8	C12—C11—H11	119.4
C6—C1—H1	119.8	C11—C12—N3	121.5 (2)
C3—C2—C1	119.1 (2)	C11—C12—C13	117.5 (2)
C3—C2—H2	120.4	N3—C12—C13	121.0 (2)
C1—C2—H2	120.4	C14—C13—C12	120.8 (2)
C2—C3—C4	121.5 (2)	C14—C13—H13	119.6
C2—C3—Cl1	120.08 (17)	C12—C13—H13	119.6
C4—C3—Cl1	118.36 (17)	C13—C14—C9	121.5 (2)
C5—C4—C3	118.8 (2)	C13—C14—H14	119.3
C5—C4—H4	120.6	C9—C14—H14	119.3
C3—C4—H4	120.6	N3—C15—H15A	109.5
C4—C5—C6	120.9 (2)	N3—C15—H15B	109.5
C4—C5—H5	119.5	H15A—C15—H15B	109.5
C6—C5—H5	119.5	N3—C15—H15C	109.5
C5—C6—C1	119.2 (2)	H15A—C15—H15C	109.5
C5—C6—C7	122.61 (19)	H15B—C15—H15C	109.5
C1—C6—C7	118.15 (19)	N3—C16—H16A	109.5
O1—C7—N1	122.92 (19)	N3—C16—H16B	109.5
O1—C7—C6	121.92 (18)	H16A—C16—H16B	109.5
N1—C7—C6	115.16 (18)	N3—C16—H16C	109.5
N2—C8—C9	120.9 (2)	H16A—C16—H16C	109.5
N2—C8—H8	119.6	H16B—C16—H16C	109.5
C9—C8—H8	119.6	H1W1—O1W—H2W1	109.6
			
C7—N1—N2—C8	−171.4 (2)	N1—N2—C8—C9	−176.96 (18)
C6—C1—C2—C3	1.9 (3)	N2—C8—C9—C10	−177.5 (2)
C1—C2—C3—C4	−0.2 (4)	N2—C8—C9—C14	4.5 (3)
C1—C2—C3—Cl1	−178.32 (17)	C14—C9—C10—C11	0.2 (3)
C2—C3—C4—C5	−1.1 (3)	C8—C9—C10—C11	−177.9 (2)
Cl1—C3—C4—C5	176.95 (17)	C9—C10—C11—C12	1.5 (3)
C3—C4—C5—C6	0.9 (3)	C10—C11—C12—N3	176.4 (2)
C4—C5—C6—C1	0.7 (3)	C10—C11—C12—C13	−1.9 (3)
C4—C5—C6—C7	179.3 (2)	C15—N3—C12—C11	151.7 (2)
C2—C1—C6—C5	−2.2 (3)	C16—N3—C12—C11	13.7 (3)
C2—C1—C6—C7	179.2 (2)	C15—N3—C12—C13	−30.0 (3)
N2—N1—C7—O1	4.3 (3)	C16—N3—C12—C13	−168.0 (2)
N2—N1—C7—C6	−175.69 (19)	C11—C12—C13—C14	0.7 (3)
C5—C6—C7—O1	−151.1 (2)	N3—C12—C13—C14	−177.7 (2)
C1—C6—C7—O1	27.4 (3)	C12—C13—C14—C9	1.0 (4)
C5—C6—C7—N1	28.8 (3)	C10—C9—C14—C13	−1.5 (3)
C1—C6—C7—N1	−152.6 (2)	C8—C9—C14—C13	176.6 (2)

### Hydrogen-bond geometry (Å, °)

**Table:**

*D*—H···*A*	*D*—H	H···*A*	*D*···*A*	*D*—H···*A*
O1W—H1W1···N3^i^	0.84	2.28	3.045 (2)	152
N1—H1N1···O1W	0.86 (1)	2.00 (1)	2.843 (3)	169 (2)
O1W—H2W1···O1^ii^	0.84	2.02	2.790 (2)	152
O1W—H2W1···N2^ii^	0.84	2.59	3.240 (3)	135
C15—H15C···Cl1^iii^	0.96	2.78	3.704 (2)	163
C1—H1···Cg1^iv^	0.93	2.97	3.621 (2)	128
C4—H4···Cg1^v^	0.93	2.89	3.565 (2)	130
C10—H10···Cg2^vi^	0.93	2.87	3.589 (2)	135
C13—H13···Cg2^vii^	0.93	2.81	3.497 (3)	131

Symmetry codes: (i) −*x*, *y*−1/2, −*z*+1/2; (ii) *x*+1, *y*, *z*; (iii) −*x*−1/2, −*y*+1, *z*−1/2; (iv) −*x*−1, *y*+1/2, −*z*+3/2; (v) −*x*, *y*+3/2, −*z*+3/2; (vi) *x*+1/2, −*y*−1/2, −*z*; (vii) *x*−1/2, −*y*+1/2, −*z*.
